# Collodion baby treated at a tertiary hospital in Tanzania: a case report

**DOI:** 10.1186/s13256-018-1912-8

**Published:** 2018-12-31

**Authors:** Evance K. Godfrey, Evelyne G Furumbe, Flora Faustine, Helga Naburi

**Affiliations:** Department of Paediatrics and Child health, School of Medicine, Muhimbili University of Health and Allied Sciences (MUHAS), 9 United Nations Road, Upanga West, P.O. Box 65001, Dar-es-salaam, Tanzania

**Keywords:** Collodion baby, Ectropium, Eclabium

## Abstract

**Background:**

The term “collodion baby” is used to describe a newborn covered with a translucent, parchment-like skin sheet. It is an extremely rare condition with an estimated incidence of 1 in 300,000 live births. Clinically, the baby will present with a collodion membrane with fissures, ectropium, eclabium, and hypoplastic digits. Shedding of the membrane increases risk of dehydration and infection.

**Case presentation:**

We present the case of an African baby girl, who died when she was 7-months old, who presented with features of collodion membrane at birth. She later developed hypernatremic dehydration and a constricted band on her lower limb that required urgent surgical release. She stayed in our hospital for 35 days; she was then discharged home after improvement for 6 months of follow-up clinics at Muhimbili National Hospital: neonatal; dermatology; ear, nose, and throat; and physiotherapy units. She died at 7 months of age.

**Conclusion:**

Despite limited resources, the early survival of these babies can be improved by providing basic care.

**Electronic supplementary material:**

The online version of this article (https://doi.org/10.1186/s13256-018-1912-8) contains supplementary material, which is available to authorized users.

## Introduction

Collodion baby (CB) is the term first used in 1884 by Hallopeau and Watelet [1–3] to describe a newborn covered with a translucent, parchment paper-like skin sheet known as a collodion [1, 4]. It is not a disease entity but it describes a phenotype of a newborn who may later develop one of a spectrum of disorders including autosomal recessive congenital ichthyosis, congenital ichthyosiform erythroderma, lamellar ichthyosis, and harlequin ichthyosis, or another form like Gaucher’s disease or self-healing CB [1, 2, 5]. The collodion membrane detaches in 2 to 4 weeks, usually revealing a permanent ichthyosis phenotype [6]. The condition is extremely rare with an estimated incidence of 1 in 300,000 births [7, 8]. There have been approximately 270 reported cases worldwide between 1884 and 2011 [4]. In addition to this we retrieved 36 cases reported from 2012 to 2016 (see Additional file 1: Table S1). Although some cases have been seen in Tanzania, none has been published. The condition is caused by various mutations. Harlequin and lamella ichthyosis is caused by a mutation of the *ABCA12* gene which encodes adenosine triphosphate-binding cassette protein transporter; as a result there is impairment of transportation of lipid glucosylceramide into lamellar granules and subsequently its absence in extracellular space [4, 9].

Clinically at delivery a CB presents with a tight skin covering the body, which may cause impaired chest expansion, ectropion, eclabium, and swollen feet with hypoplastic digits. Due to the impaired barrier function of the skin, CBs are at increased risk of infection, fluid loss, dehydration, electrolyte imbalance, and body temperature instability. The primary aim of treatment is to eliminate scaling and to reduce xerosis without causing excessive irritation; this can be achieved by daily bathing with water and frequent applications of a substantial amount of mild emollients such as petroleum jelly. Comprehensive treatment to improve long-term survival involves a multidisciplinary approach including neonatal, dermatological, ophthalmological otorhinolaryngology, plastic surgery care, and physiotherapy.

## Case presentation

We report a case of an African baby girl, who died at 7-months old, who was first seen in our hospital during the first 6 hours of life; she presented with a generalized cracked skin all over her body. She was delivered at 37 weeks’ gestation by caesarean section due to breech presentation; she weighed 2.5 kg, her length was 51 cm, and head circumference was 35 cm. Her Apgar score was 8 and 10 at first and fifth minutes, respectively. During pregnancy, her mother received reasonable prenatal care including screening for HIV and syphilis, which were all negative; however, a prenatal ultrasound was not done. She had no history of taking alcohol, drug abuse, or use of other medicines apart from hematenics and malaria prophylaxis.

The baby is the first born to the 24-year-old mother and 27-year-old father; there is no history of consanguinity or similar dermatological condition in the family.

On examination she was alert, her temperature was 37 °C, she had no difficulty in breathing and she was saturating well in room air, her respiratory rate was 49 breaths/minute, and her heart rate was 123 beats/minute. She was noted to have a thick parchment-like skin all over her body with peeling and varying degree of fissures, eversion of eyelids (ectropion), her mouth was wide open like a fish (eclabium), and she had hypoplastic digits and nail dystrophy, and swollen feet and hands. She also had scaling alopecia and her ear canals were filled with glue-like material. She also had a restricted range of movement in her extremities especially extension of both upper and lower limbs. The rest of the systemic examination was essentially normal.

Initial baseline investigations taken 6 hours post admission revealed random blood sugar 8.4 mmol/dl, serum sodium 154mmlo/l, creatinine 138.3 μmol/l, and normal potassium. Laboratory tests repeated 2 days later revealed normal creatinine, blood glucose, and sodium. A sepsis workup was performed on the day of admission, a report from blood culture and swab from a fissure showed no bacteria growth, and C-reactive protein (CRP) as well as complete blood count (CBC) were normal. Genetic testing was not done because it is not available in our setting.

Empirical antibiotic therapy of Ampiclox (ampicillin and cloxacillin) 125 mg 12 hourly and gentamycin 12.5 mg once daily was initiated and was continued for 5 days while awaiting results of the sepsis screening test. In addition, intravenously administered fluid (dextrose normal saline) at 175 ml per 24 hours was administered on the first day; on the second day, orogastric tube feeding was started, the baby received 25 ml (increased by 1 ml/day) of expressed breast milk every 3 hours until her seventh day of life when she was able to suck from the breast.

In the first few days of life her eyes were covered by gauze soaked in normal saline and from the third day chloramphenicol eye ointment was applied twice daily to keep her eyes lubricated. Conservative skin management was started using Vaseline (petroleum jelly), which was applied to her skin every 3 hours; strict infection prevention control that included isolation was adhered to and the baby was monitored for signs of hypothermia, dehydration, and sepsis.

**Fig. 1:**
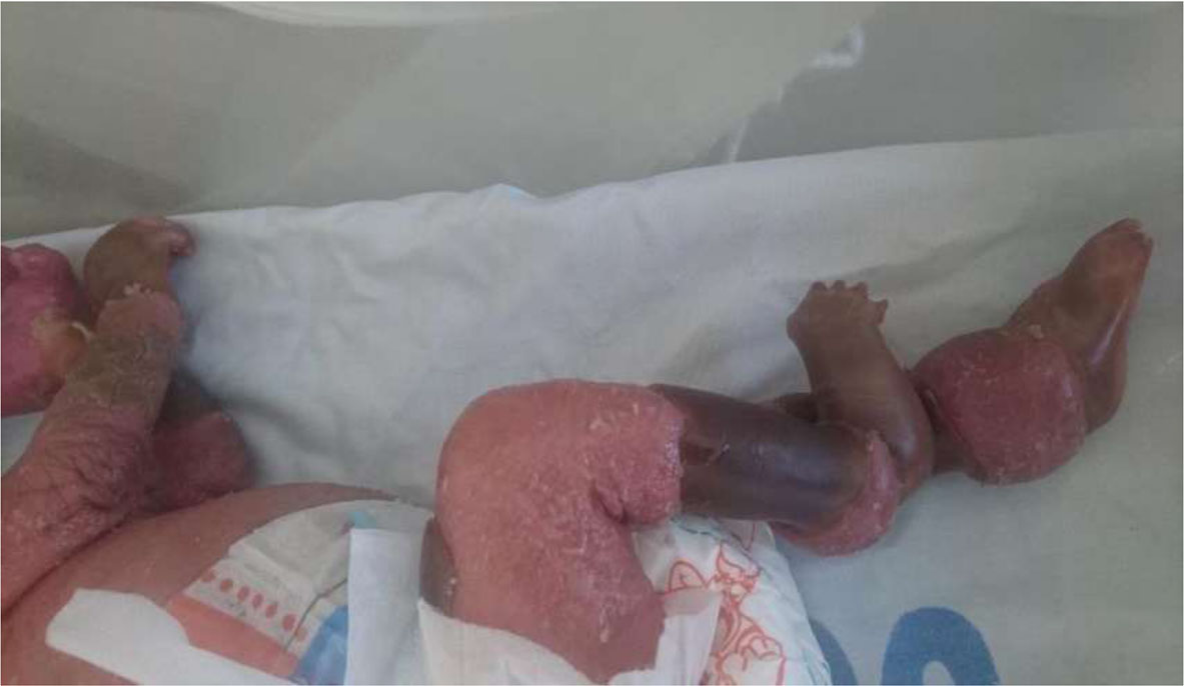
Swollen limbs. Picture taken on 24th day of life showing constricted band on both limbs with markedly swollen limbs

While the baby was still in our hospital, by the 24th day of life, a significant shedding of the collodion skin had taken place. During this time she was noted to have a constricted band on her lower limb necessitating band release (Fig. 1).

A dermatologist, otorhinolaryngologist, ophthalmologist, and pediatric surgeon were consulted and provided their multidisciplinary expertise for both short-term and long-term management of this newborn. To allow for adequate chest expansion and to prevent respiratory insufficiency on the second day of life, a pediatric surgeon made an incision along the collodion membrane on the anterior chest wall (sternal region).

A multidisciplinary team reached a decision to discharge the baby on her 35th day of life. The family was advised that the baby should attend follow-up clinics: neonatal; ophthalmology; dermatology; physiotherapy; and ear, nose, and throat (ENT).

**Fig. 2:**
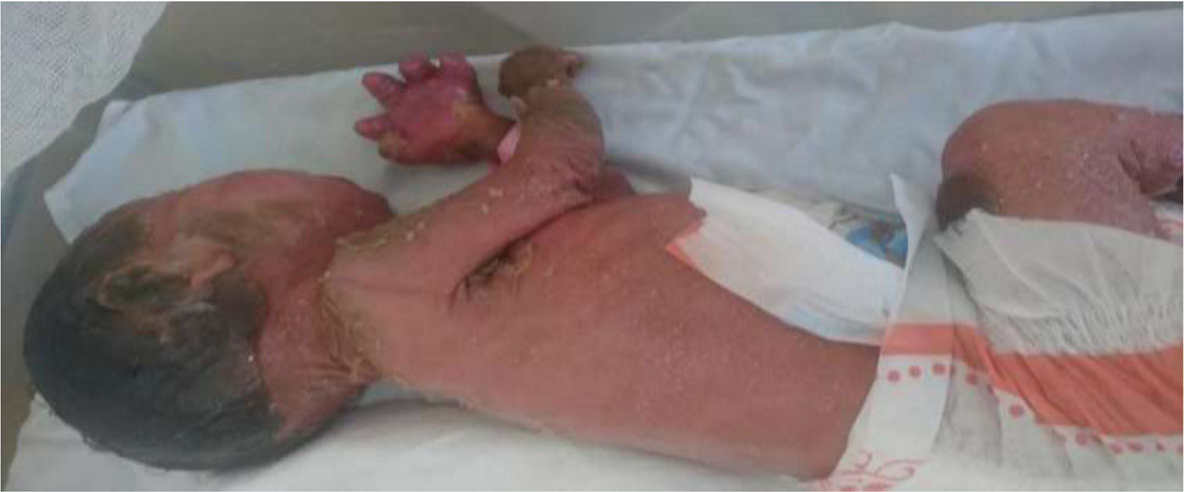
Physical findings on day 35. Picture taken on 35th day of life showing scaly skin on the trunk after a significant shedding off of the collodion membrane. The picture also shows the limited extension of the elbow joint

By the time she was discharged, there was some improvement in ectropion, eclabium, and there was relief in contracture of her fingers and toes; however, extension of knee and elbow joints were still limited (Fig. 2).

The mother and family were given counselling about the diagnosis, which involved clinicians, nurses, and a social worker; this was started on the day of admission and continued throughout the hospital stay. In addition to the care provided in the hospital, during the hospital discharge, the mother was advised on the care of the baby at home including breastfeeding, skin care, eye care, and care of joints. The baby received initial vaccines before discharge; continuation with the remaining vaccines according to the immunization and vaccine development program in Tanzania was advised.

The baby’s weight initially decreased to 2.25 kg during the first 2 weeks, then she started to gain weight; on discharge she weighed 3.2 kg and 2 weeks later when she came for a follow-up clinic she weighed 3.4 kg. She continued attending follow-up clinic for the first 6 months; however, she suddenly died at home at 7 months of age.

## Discussion

CB is a term used to describe a newborn covered with a thick tight membrane all over the body with fissures; this membrane subsequently sheds off [2]. It is not a single disease entity, but rather a phenotype that eventually demonstrates one of a spectrum of disorders including autosomal recessive congenital ichthyosis, lamellar ichthyosis, and harlequin ichthyosis, or less commonly may heal completely (self-healing), especially in those presenting with a membrane restricted to the extremities [2, 5].

Although we had no capacity to do genetic testing to determine the phenotype, the clinical presentation of this patient suggests a harlequin or lamellar ichthyosis. This is because of the presence of collodion membrane at birth with eclabium, ectropion, scaly alopecia, edematous feet, and nail hypoplasia with subsequently scaly erythematous skin.

Harlequin ichthyosis is the most severe form of a rare, autosomal recessive congenital ichthyosis. It was previously considered to be associated with poor survival, but in the medical literature survival beyond 7 years of age has been reported [10].

Among the known complications of this condition that increase the risk of mortality are infection, fluid loss, dehydration, electrolyte imbalance, and body temperature instability.

Our patient presented with mild hypernatremia and high serum creatinine, these investigation results were before administration of intravenously administered dextrose normal saline, which resolved within 2 days after adequate hydration. Severe hypernatremia with uremia which responded well to fluid correction has been reported [8]. Thus prompt correction of dehydration is essential as these babies are at increased risk of fluid loss through bare skin and loss to surroundings if they are not kept in a humidified room [3, 11]. In our case a special humidifier was not used but the baby was covered with gauze soaked in a lukewarm saline to prevent fluid loss through bare skin.

In an ideal setting, these babies need to be admitted to a neonatal intensive care unit and cared for in high humidity incubators [11]. However, this is challenging in resource-limited settings where the newborn unit may not be able to fulfill these requirements. However, with close monitoring, proper hygiene, and isolation to reduce risk of infection, the survival of these babies can be improved. In our case, there were no signs of sepsis. A literature review revealed that there are babies who died within a few days due to sepsis and septic shock [12, 13], so we considered the possible risk of infections in this baby because of the impaired barrier function of her skin and increased susceptibility to *Staphylococcus aureus*, *Streptococcus pyogenes*, and *Klebsiella* species infections [10, 12–15]. Thus we initiated the baby on antibiotics by intravenously administering Ampiclox (ampicillin and cloxacillin) and gentamycin until sepsis was excluded by the laboratory results of CRP, CBC, and blood culture.

Other known complications, such as respiratory failure and temperature deregulations [11, 13], were not observed in this case.

Another important aspect of care of these babies is eye care, because the tight membrane does not allow the eye to close normally, and the conjunctiva is exposed, which can lead to exposure keratitis [15]. The long-term complications include hyperopia and anisometropia which may necessitate wearing of glasses [14]. Persistence of ectropion by age of 6 months may need a surgical correction [11]. In the case presented here, the ectropion had some improvement in the first month of life; the baby was closely monitored by ophthalmologists, who provided regular follow-up eye care.

These babies are at increased risk of developing conductive hearing loss [1, 7, 11]. A case of a baby with ichthyosis who was diagnosed as having conductive hearing loss on 83rd day of life has been reported; the problem persisted for the whole duration of the 2-year follow-up [15]. We cannot predict the hearing outcome of the baby we have presented because a hearing test was not conducted. The baby presented with excessive glue-like material in her ears and an ENT specialist recommended daily application of normal saline and weekly follow-up visits; a hearing assessment would have been conducted when appropriate.

The patient presented here developed a constricted band, which impaired blood flow to her distal extremities, which caused swelling of her limbs, necessitating urgent surgical release. A delay in the release the band could have caused acute compartment syndrome and gangrene to the distal limbs [7, 11, 16], which could result in loss of the limb.

We could not make a definitive diagnosis to determine the phenotype, since it needs genetic testing of both parents and the baby, which is currently unavailable in our hospital.

## Conclusion

Despite limited resources, the early survival of these babies can be improved by providing basic care, which includes: proper hydration; infection control; and proper skin, eye, and ear care.

## Additional file

Additional file 1: **Table S1.** shows reported cases of collodion babies since 2012. (DOCX 24 kb)

## Abbreviations

CB: Collodion baby
CBC: Complete blood count
CRP: C-reactive protein
ENT: Ear, nose, and throat
