# Supplementary material for: Collodion baby treated at a tertiary hospital in Tanzania: a case report
Source: J Med Case Rep. 2018 Dec 31;12:385. doi: 10.1186/s13256-018-1912-8 (PMC6311898; doi:10.1186/s13256-018-1912-8)
Supplement: Supplementary file 1 — Table S1. shows reported cases of collodion babies since 2012. (DOCX 24 kb) [file 13256_2018_1912_MOESM1_ESM.docx]

| Author’s name | Year | Country | Age at diagnosis | Age at last contact with author |
| --- | --- | --- | --- | --- |
| HA Obu | 2013 | NIGERIA (4 cases ) | Within 24hrs | 1^st^ case discharged2^nd^ case died3^rd^ on 19^th^ day4^th^ on 11^th^ day |
| Kuponiyi OT | 2016 | NIGERIA 1 CASE | Within 24hrs | 43 days |
| Houssem Ragmoun | 2017 | TUNISIA | Within 12hrs | Died within 24hrs |
| [Priyanka Srivastava](https://www.ncbi.nlm.nih.gov/pubmed/?term=Srivastava%20P%5BAuthor%5D&cauthor=true&cauthor_uid=27504372) | 2016 | INDIA | Within 24hrs | Loss to follow up |
| Sharja Phuljhele | 2015 | INDIA | 2^nd^ day | Not documented |
| Bhardwaj U | 2012 | NEPAL | 4th day | 15 days |
| [Anwar A Mithwani](https://www.ncbi.nlm.nih.gov/pubmed/?term=Mithwani%20AA%5BAuthor%5D&cauthor=true&cauthor_uid=24717853) | 2014 | INDIA | Within 24hours | 7years |
| Kumar P | 2015 | INDIA | 3rd day | Loss to follow up at 17 days |
| [Bruno Ferrari](https://www.ncbi.nlm.nih.gov/pubmed/?term=Ferrari%20B%5BAuthor%5D&cauthor=true&cauthor_uid=28492026) | 2016 | ARGENTINA 5 CASES | Within 24hrs | 1^st^ 4 weeks2^nd 21 days^  ^3rd^ weeks 24^th^ 1 month5^th^ 2 weeks |
| Vesna Stojanovic | 2014 | SERBIA | Soon after delivery | 1 year |
| Shah Farhat A, | 2017 | IRAN | Soon after delivery | 16 days |
| [Deren Özcan](https://www.ncbi.nlm.nih.gov/pubmed/?term=%26%23x000d6%3Bzcan%20D%5BAuthor%5D&cauthor=true&cauthor_uid=24371398) | 2013 | TURKEY | Soon after delivery | 3years |
| Selvi Gulasi1 | 2016 | IRAN | Soon after delivery | 28 days |
| Janardhan Bommakanti | 2015 | INDIA 2 cases | 1^st^ fourth day2^nd^ 6^th^ day of life | 2^nd^ and 3^rd^ day respectively |
| Shibani Pal | 2015 | INDIA | Soon after delivery | Died on 5^th^ day of life |
| Ali Irfan Güzel | 2014 | Turkey | Immediate after delivery | Died within 24hrs |
| Jakeer Shaik | 2015 | INDIA | Soon after delivery | Not mention |
| Sweta S. Kumar | 2015 | INDIA | 3month | Refered to another center at 3month |
| Sanjoy Chowdhury | 2017 | INDIA | Soon | Died within 24hours |

| Rakesh Chakinala1 |  | INDIA | Soon after delivery | Admitted on 18^th^ julyDischrged 13^th^ july­­­ |
| --- | --- | --- | --- | --- |
| Morazan AFD | 2017 | Korea | Soon after delivery | refered to higher facility |
| Michelle A | 2015 | USA | Soon after delivery | 16^th^ day |
| Arun Kumar | 2013 | India | Soon after delivery | Passed away on the 2^nd^ day |
| D. Y Shrikhande | 2014 | INDIA | Soon after delivery | Passed away 5^th^ day |
| Pilar Puy | 2013 | CANADA | 6 days | Within neonatal period |
| S.M. Rawlani, | 2013 | INDIA | 8 years | 8years |
| MK Hassan | 2016 | BAGLADESH | Within 24hrs | 1week of life |
| Laura M Chang | 2014 | USA | Soon after delivery | 9 month |
